# Gene expression signature of estrogen receptor α status in breast cancer

**DOI:** 10.1186/1471-2164-6-37

**Published:** 2005-03-11

**Authors:** Martín C Abba, Yuhui Hu, Hongxia Sun, Jeffrey A Drake, Sally Gaddis, Keith Baggerly, Aysegul Sahin, C Marcelo Aldaz

**Affiliations:** 1 Department of Carcinogenesis, The University of Texas M.D. Anderson Cancer Center, Science Park-Research Division, Smithville, Texas, USA; 2 Department of Biostatistics, The University of Texas M.D. Anderson Cancer Center, Houston, Texas, USA; 3 Department of Pathology, The University of Texas M.D. Anderson Cancer Center, Houston, Texas, USA

## Abstract

**Background:**

Estrogens are known to regulate the proliferation of breast cancer cells and to modify their phenotypic properties. Identification of estrogen-regulated genes in human breast tumors is an essential step toward understanding the molecular mechanisms of estrogen action in cancer. To this end we generated and compared the Serial Analysis of Gene Expression (SAGE) profiles of 26 human breast carcinomas based on their estrogen receptor α (ER) status. Thus, producing a breast cancer SAGE database of almost 2.5 million tags, representing over 50,000 transcripts.

**Results:**

We identified 520 transcripts differentially expressed between ERα-positive (+) and ERα-negative (-) primary breast tumors (Fold change ≥ 2; p < 0.05). Furthermore, we identified 220 high-affinity *Estrogen Responsive Elements *(EREs) distributed on the promoter regions of 163 out of the 473 up-modulated genes in ERα (+) breast tumors. In brief, we observed predominantly up-regulation of *cell growth *related genes, *DNA binding *and *transcription factor activity *related genes based on Gene Ontology (GO) biological functional annotation. GO terms over-representation analysis showed a statistically significant enrichment of various transcript families including: *metal ion binding *related transcripts (p = 0.011), *calcium ion binding *related transcripts (p = 0.033) and *steroid hormone receptor activity *related transcripts (p = 0.031). SAGE data associated with ERα status was compared with reported information from breast cancer DNA microarrays studies. A significant proportion of ERα associated gene expression changes was validated by this cross-platform comparison. However, our SAGE study also identified novel sets of genes as highly expressed in ERα (+) invasive breast tumors not previously reported. These observations were further validated in an independent set of human breast tumors by means of real time RT-PCR.

**Conclusion:**

The integration of the breast cancer comparative transcriptome analysis based on ERα status coupled to the genome-wide identification of high-affinity EREs and GO over-representation analysis, provide useful information for validation and discovery of signaling networks related to estrogen response in this malignancy.

## Background

Estrogen plays essential roles in the development, growth control and differentiation of the normal mammary gland. However, it is well documented that endogenous estrogens are powerful mitogens critical for the initiation and progression of human breast and gynecological cancers [1]. This cell proliferation signal is mediated by the estrogen receptors (ER), members of the nuclear receptor family that function both as signal transducers and transcription factors to modulate expression of target genes [2]. There are two main subtypes of estrogen receptors: ERα and ERβ that generally can form homo- and heterodimers before binding to DNA. Although the DNA binding domains of these receptors are very similar, the overall degree of homology is low [3].

Transcriptional regulation of target genes in response to 17β-estradiol (E_2_) is mediated by two main mechanisms. In one, the E_2_-ER complex binds to a specific DNA sequence called the estrogen response element (ERE), this receptor-ligand DNA bounded complex interacts with co-regulatory proteins, promoting chromatin remodeling and bridging with the general gene transcription machinery thus resulting in transcription initiation [4]. Alternatively, the ligand-ER complex can interact with other DNA-bound transcription factors that in turn bind DNA sequences (e.g. via AP1, SP1 complexes) [5,6]. ERα and ERβ have different affinities for different response elements and exhibit distinct transcriptional properties. Additionally, E_2 _also exerts rapid, non-genomic effects attributed to cell membrane-initiated signaling [7].

Approximately two-thirds of all breast cancers are ERα (+) at the time of diagnosis and expression of this receptor is determinant of a tumor phenotype that is associated with hormone-responsiveness. Patients with tumors that express ERα have a longer disease-free interval and overall survival than patients with tumors that lack ERα expression [8]. However, the association between ERα expression and hormonal responsiveness is not perfect: approximately 30% of ERα-positive tumors are not hormone-responsive while 5–15% of ERα-negative tumors respond to hormonal therapy [9]. The molecular basis for the association between ERα expression, hormonal responsiveness and breast cancer prognosis remains unclear.

Several studies have been carried out using cDNA and oligonucleotide microarrays identifying breast cancer subclasses possessing distinct biological and clinical properties [10-13]. Among the distinctions made to date, the clearest separation was observed between ERα (+) and ERα (-) tumors [10-15]. It has been suggested that there are sets of genes expressed in association with ERα that could play an important role in determining the hormone-responsive breast cancer phenotype [16]. ERα is obviously likely to be important for the E_2 _induced proliferative response predominantly via the regulation of estradiol-responsive genes. Nevertheless, the expression of additional subsets of genes not necessarily directly regulated by estrogen may also be fundamental in defining the breast cancer hormone-responsive phenotype.

To further elucidate the molecular basis of estrogen-dependent breast carcinogenesis, we here report a comparative transcriptome profiling of invasive breast tumors based on ERα status obtained by SAGE. The SAGE method provides a statistical description of the mRNA population present in a cell without prior selection of the genes to be studied, and this constitutes a major advantage [17]. The breast cancer SAGE comparative analysis was combined with promoter sequence analysis of genes of interest using high-throughput methods of high-affinity ERE identification. In order to have an even more comprehensive picture we also performed a cross-platform comparison between SAGE and DNA microarray studies.

## Results and discussion

### Biomarkers of ERα status in breast carcinomas

The primary goal of our study was to identify the most commonly deregulated genes in invasive breast carcinomas related to ERα status. To this end SAGE data was obtained from a set of primary breast carcinomas. Thus, a breast cancer SAGE database of almost 2.5 million tags was analyzed, representing over 50,000 tag species. We performed a comprehensive evaluation and comparison of gene expression profiles using a recently developed supervised method [18], to identify the most representative differentially expressed transcripts between tumors groups, *i.e. *ERα (+) vs. ERα (-) breast tumors.

This statistical analysis revealed 520 genes differentially expressed (Fold change ≥ 2; p < 0.05) between ERα (+) and ERα (-) primary breast carcinomas (see additional data file 1). Among the 520 transcripts, 473 were up-modulated and 47 were down-modulated transcripts in ERα (+) tumors.

The most commonly over-expressed transcripts in ERα (+) tumors were: *trefoil factor 1 *(*TFF1/pS2*), *synaptotagmin-like 4 *(*SYTL4*), *regulating synaptic membrane exocytosis 4 (RIMS4), dual specificity phosphatase 4 *(*DUSP4*), *chromosome 1 open reading frame 34 *(*C1orf34*), *necdin homolog *(*NDN*), *n-acetyltransferase 1 *(*NAT1*) and *caspase recruitment domain family 10 *(*CARD10*) (Table 1 and additional data file 1).

**Table 1:** Most highly up-modulated transcripts in ERα (+) breast carcinomas identified by SAGE.

**Gene name**	**Tag**	**Locus Link**	**Fold change (*p *value)**	**Frequency^#^**
**Cell proliferation related**				
TFF1* *(trefoil factor 1)*	CTGGCCCTCG	7031	51.4 (0.0016)	15/18 (83%)
***DUSP4 ****(dual specificity phosphatase 4)*	CGGGCAGAAA	1846	14.7 (0.0016)	14/18 (78%)
NDN* *(necdin homolog)*	ACCTTGCTGG	4692	13.3 (0.0026)	11/18 (61%)
***HDGFRP3 ****(hepatoma-derived growth factor)*	TGTAAAGTTT	50810	9.8 (0.0019)	12/18 (67%)
TSPAN1* *(tetraspan 1)*	GGAACTGTGA	10103	9.5 (0.0017)	15/18 (83%)
***SEP6 ****(septin 6)*	TCAATTTTCA	23157	7.6 (0.0044)	12/18 (67%)
DHX34* *(DEAH box polypeptide 34)*	GTTGCTCACT	9704	7.1 (0.0129)	9/18 (50%)

**Apoptosis related**				
CARD10* *(caspase recruitment domain family)*	AGAATGTACG	29775	11.1 (0.0030)	15/18 (83%)

**Signal transduction related**				
SYTL4* *(synaptotagmin-like 4)*	TATGTGTGCT	94121	28.0 (0.0003)	15/18 (83%)
ECM1* *(extracellular matrix protein 1)*	ACTGCCCGCT	1893	10.1 (0.0175)	13/18 (72%)
LEPR* *(leptin receptor)*	AAAGTTTGAG	3953	9.8 (0.0302)	10/18 (55%)
***PTGES ****(prostaglandin E synthase)*	TGAGTCCCTG	9536	8.0 (0.0168)	8/18 (44%)
***SCUBE2 ****(signal peptide, CUB domain EGF-like 2)*	TCAGCACAGT	57758	7.5 (0.0024)	14/18 (78%)
ADORA2A* *(adenosine A2a receptor)*	TGCTGAGTAG	135	7.1 (0.0460)	11/18 (61%)
***ITGBL1 ****(integrin beta-like 1)*	CATATTCACA	9358	7.1 (0.0159)	8/18 (44%)

**Regulation of transcription related**				
***ESR1 ****(estrogen receptor 1)*	AGCAGGTGCC	2099	9.8 (0.0000)	18/18 (100%)
***TCEAL1 ****(transcriptional elongation factor A)*	AAAGATGTAC	9338	9.8 (0.0014)	13/18 (72%)
***ZNF14 ****(zinc finger protein 14)*	TAAACAGCCC	7561	8.4 (0.0023)	13/18 (72%)
ZNF38* *(zinc finger protein 38)*	CCAGCATTAC	7589	7.6 (0.0051)	10/18 (55%)
HIF1AN* *(hypoxia-inducible factor 1α subunit inhibitor)*	CCTGAGTGCG	55662	7.1 (0.0094)	10/18 (55%)
***HOXC13 ****(homeo box C13)*	TTTTTAAAAT	3229	7.1 (0.0157)	9/18 (50%)

**Cytoskeleton**				
***MAPT ****(microtubule-associated protein tau)*	GTAGACTCGC	4137	9.8 (0.0085)	9/18 (50%)
***MYLIP ****(myosin regulatory light chain interacting)*	TTTTCCACTC	29116	9.3 (0.0036)	11/18 (61%)

**Metabolism and Miscelaneous**				
***RIMS4 ****(regulating synaptic membrane exocytosis)*	TTGAAATTAA	140730	24.9 (0.0378)	8/18 (44%)
***NAT1 ****(N-acetyltransferase 1)*	TATCTTCTGT	9	11.7 (0.0385)	15/18 (83%)
ATP6V1B1* *(ATPase, H+ transporting)*	CCTCCCCCTC	525	10.7 (0.0111)	10/18 (55%)
***JDP1 ****(J domain containing protein 1)*	TCTGTGAATT	56521	10.0 (0.0035)	12/18 (67%)
***CHST11 ****(carbohydrate sulfotransferase 11)*	AACCTTCCTC	50515	9.8 (0.0009)	13/18 (72%)
***CILP ****(nucleotide pyrophosphohydrolase)*	GTTTTGCCCA	8483	9.3 (0.0054)	14/18 (78%)
***ABCA3 ****(ATP-binding cassette sub-family A)*	GTAGTCACCG	21	8.9 (0.0149)	10/18 (55%)
***SEC14L2***	GGAAGGCGGC	23541	8.7 (0.0487)	9/18 (50%)
ANXA9* *(annexin A9)*	ACATCCGAGG	8416	8.4 (0.0145)	10/18 (55%)
***KCTD3 ****(K channel tetramerisation domain 3)*	ATAATTAAAT	51133	8.4 (0.0001)	17/18 (94%)
***SFRS7 ****(splicing factor)*	TAGCTAATAT	6432	8.0 (0.0031)	12/18 (67%)
SNRPA* *(small nuclear ribonucleoprot. polypep. A)*	AAGATCTCCT	6626	7.6 (0.0009)	15/18 (83%)
***NNMT ****(nicotinamide N-methyltransferase)*	CCTGCAATTC	4837	7.6 (0.0120)	10/18 (55%)
***SLC1A4 ****(solute carrier family 1 member 4)*	GACTCACAGG	6509	7.6 (0.0254)	9/18 (50%)
***TIPARP ****(TCDD-inducible polymerase)*	AAATGGCCAA	25976	7.6 (0.0051)	10/18 (55%)
***SLC7A2 ****(solute carrier family 7 member 2)*	CACTGACAGC	6542	7.3 (0.0190)	11/18 (61%)
GA* *(liver mitochondrial glutaminase)*	CTGCTGCTAC	27165	7.1 (0.0126)	9/18 (50%)

**Function unknown**				
***C1orf34***	AGGATGTACA	22996	13.3 (0.0025)	14/18 (78%)
***SMILE ****(hypothetical protein FLJ90492)*	TAGAGAGTTT	160418	11.1 (0.0004)	15/18 (83%)
***RHBDL4 ****(rhomboid, veinlet-like 4)*	TTGTTTCTAA	162494	10.7 (0.0099)	9/18 (50%)
***KIAA0882***	GTCTCATTTC	23158	10.1 (0.0007)	18/18 (100%)
C20orf103*	TTTAGTGATT	24141	9.3 (0.0277)	10/18 (55%)
***FLJ33387***	GCAGGGAGAG	161145	9.3 (0.0118)	10/18 (55%)
***TRALPUSH***	GTTTCCAGAG	116931	8.9 (0.0458)	9/18 (50%)
KIAA0980*	TGGTGCTTCC	22981	7.6 (0.0096)	11/18 (61%)
***C10orf32***	AGTCTGTTGT	119032	7.3 (0.0002)	15/18 (83%)
***FLJ13611***	TAATCACACT	80006	7.1 (0.0069)	10/18 (55%)

* Genes with known or putative high-affinity EREs mapping in the vicinity of the TSS.

^# ^Transcripts tags changing > 2-fold when compared with the average expression of ER (-) tumors in at least 8 of 18 (44%) ERα (+) invasive carcinomas SAGE libraries.

For the whole list of ERα associated transcripts see additional data file 1.

**Figure 1:**
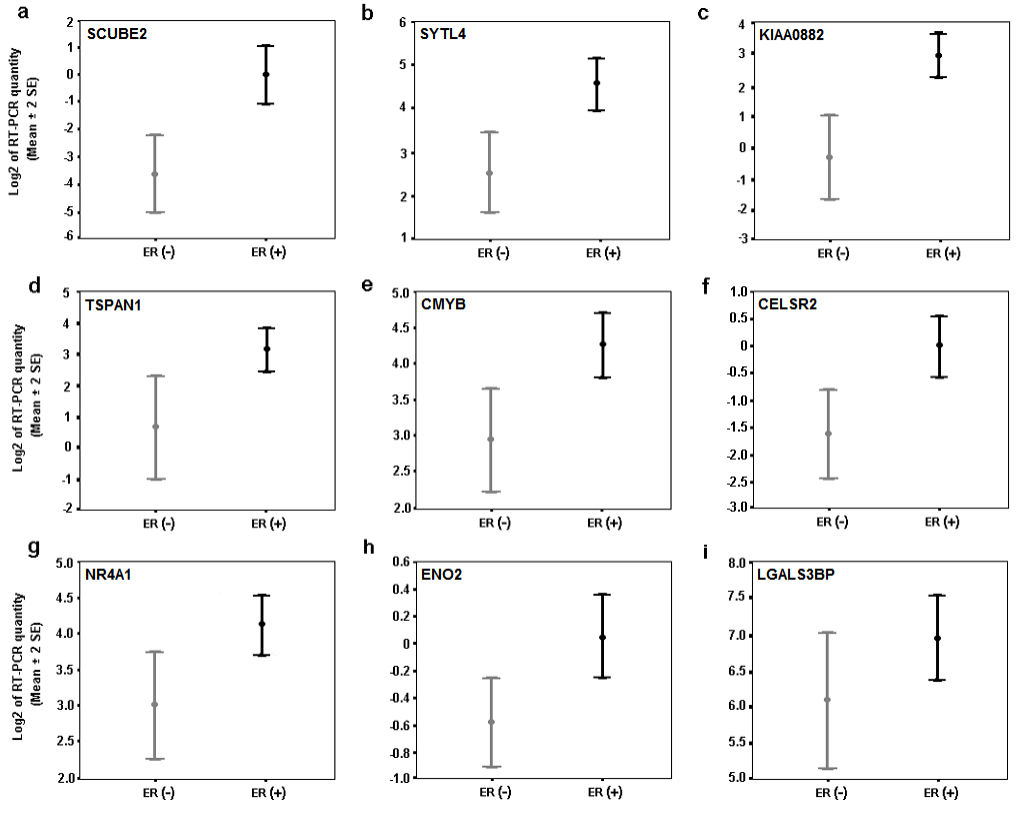
**Real time RT-PCR validation of nine over-expressed genes in 36 invasive breast carcinomas. a) ***SCUBE2 *(p = 0.0001); **b) ***SYTL4 *(p = 0.0005); **c) ***KIAA0882 *(p = 0.0005); **d) ***TSPAN1 *(p = 0.001); **e) ***CMYB *(p = 0.002); **f) ***CELSR2 *(p = 0.011); **g) ***NR4A1 *(p = 0.029); **h) ***ENO2 *(p = 0.033); **i) ***LGALS3BP *(p = 0.079). Mean ± 2 Standard Error based on Log2 transformation of real time RT-PCR values of the assayed gene relative to 18S rRNA used as normalizing control.

To validate novel ERα associated genes detected by SAGE not reported in other studies, we performed Real Time RT-PCR analysis of representative transcripts in an independent set of 36 invasive ductal breast carcinomas. In agreement with our SAGE analysis, we detected statistical differences in the over-expression of 8 out of 9 evaluated transcripts in ERα (+) breast tumors including: *signal peptide CUB domain EGF-like 2 *(*SCUBE2*) (p = 0.0001), *SYTL4 *(p = 0.0005), *KIAA0882 protein *(p = 0.0005), *tetraspan 1 *(*TSPAN1*) (p = 0.001), *myeloblastosis viral oncogene homolog *(*C-MYB*) (p = 0.002), *epidermal growth factor-like *2 (*CELSR2*) (p = 0.011), nuclear *receptor subfamily 4 *(*NR4A1*) (p = 0.029), and *enolase 2 *(*ENO2*) (p = 0.033) (Figure 1). A trend of borderline significance was detected for the *lectin galactoside-binding protein *(*LGALS3BP*) (p = 0.079) transcript (Figure 1).

*SCUBE2 *(also known as *EGF-like 2 or CEGP1*) encodes a secreted and cell-surface protein containing EGF and CUB domains that defines a novel gene family [19]. The epidermal growth factor (EGF) motif is found in many extracellular proteins that play an important role during development, functioning as secreted growth factors, transmembrane receptors, signaling molecules, and important components of the extracellular matrix. The CUB domain is found in several proteins implicated in the regulation of extracellular process such as cell-cell communication and adhesion [20]. Expression of *SCUBE2 *has been detected in vascular endothelium and may play important roles in development, inflammation and perhaps carcinogenesis [19].

The *CELSR2 *gene (also known *EGFL2*) encodes a protein member of the nonclassic-type cadherins (flamingo subfamily). These 7-pass transmembrane proteins have nine cadherin domains, seven-epidermal growth factor-like repeats and two laminin A G-type repeats [21]. It is postulated that these proteins are receptors involved in cell adhesion and receptor-ligand interactions [21] playing a role in developmental processes and cell growth/ maintenance in epithelial and neuronal cells [22,23].

*SYTL4 *(also known as *granuphilin-a *or *SLP4*) contains an N-terminal Slp homology domain (SHD) than can specifically and directly bind the GTP-bound form of Rab27A, a small GTP-binding protein involved in granule exocytosis in cytotoxic T lymphocytes [24]. We determined that over-expression of *SYTL4 *is associated with ERα (+) tumors (Figure 1b). However, the potential role of this gene in breast carcinogenesis remains unknown.

*ENO2 *(also known as *NSE/neuron-specific gamma enolase*) encodes one of three enolase isoenzymes found in mammals. This isoenzyme was described to be expressed in cells of neuronal origin. Interestingly, in a recent report Hao *et al*. (2004) showed high expression of *ENO2 *transcripts in breast cancer lymph node metastases when compared with primary breast tumors [25].

The *TSPAN1 *gene (also known as *tetraspanin *or *NET1*) encodes a cell-surface protein member of the transmembrane 4 superfamily (*TM4SF*), involved in the regulation of cell development, activation, growth and motility. A number of tetraspanins were described as tumor-specific antigens, and it was suggested that the function of some TM4SF proteins may be particularly relevant to tumor cell metastasis [26]. Sugiura and Berditchevski (1999) observed that *TSPAN1 *protein complexes may control the invasive migration of tumor cells and contribute to ECM-induced production of MMP2 in breast cancer cell line [27].

*NR4A1*, a nuclear receptor subfamily 4, group A gene (also known as *steroid receptor TR3 *or *NUR77*) encodes an orphan member of the steroid-thyroid hormone-retinoid receptor superfamily whose members mainly act as transcriptional factors to positively or negatively regulate gene expression and play roles in regulating growth and apoptosis [28,29]. A role for *NR4A1 *in cell proliferation has been previously reported. It was shown that its expression is rapidly induced by various mitogenic stimuli such as: serum growth factor, epidermal growth factor and fibroblast growth factor [28].

Taken together, the genes that we identified and validated appear to be involved in signaling pathways related to cell proliferation, invasion and metastatic processes, but their exact role in breast carcinogenesis remains to be elucidated.

### Gene Ontology analysis

**Figure 2:**
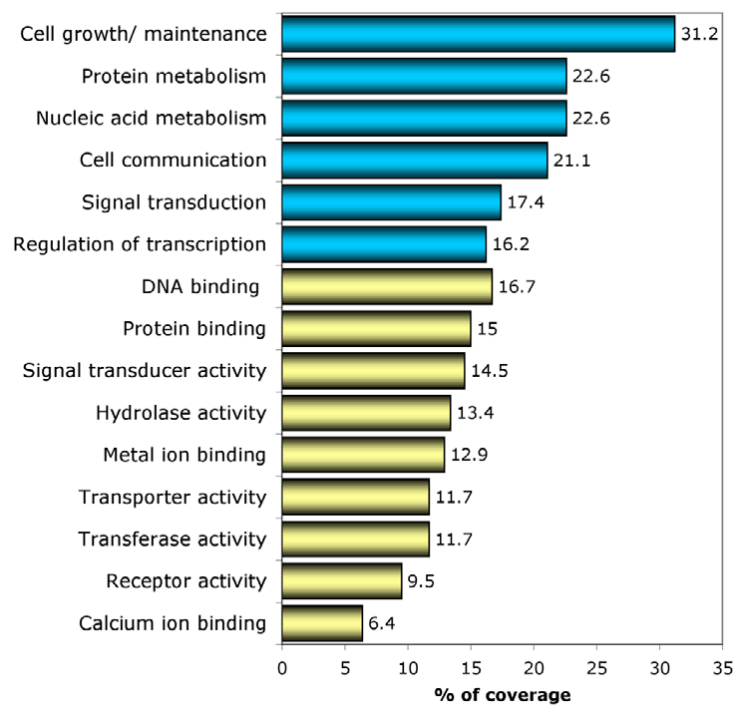
**GO classification of the ERα associated genes identified by SAGE. **Percent of coverage representing the percentage of genes annotated with a specific GO term related to Biological Processes (blue bars) and Molecular Function (yellow bars).

Classification of genes based on Gene Ontology (GO) terms is a powerful bioinformatics tool suited for the analysis of DNA microarray and SAGE data. Analysis of GO annotation allows one to identify families of genes that may play significant roles related to specific molecular or biological processes in expression profiles [30]. We used the *Expression Analysis Systematic Explorer *software (*EASE*) [31] to annotate the 520 deregulated genes according to the information provided by the GO Consortium [30]. The GO database provided annotation for 80% (419 out of 520) of the genes identified by SAGE. Results of this analysis are shown in Figure 2 and in detail in additional data file 2.

We observed that 31% of ERα associated transcripts are involved in biological processes related to *cell growth and/or maintenance*, 21% are related to *cell communication*, and 16% are related to *regulation of transcription*. Approximately 16% of these deregulated genes are related to molecular functions associated with *DNA binding *and more specifically with *transcription factor activity *(10%) (Figure 2). Interestingly, using the enrichment GO terms analysis, we identified statistical significant over-representation of specific groups of proteins including: *metal ion binding proteins *(54 hits out of 419 annotated genes; p = 0.011), *calcium ion binding proteins *(27 hits out of 419; p = 0.032) and *steroid hormone receptor activity related proteins *(6 hits out of 419; p = 0.031) (additional data file 2). The GO cluster related to *steroid hormone receptor activity proteins *includes: *estrogen receptor 1 *(*ESR1, i.e. ERα*), *androgen receptor *(*AR*), *hydroxysteroid 17-β dehydrogenase 4 *(*HSD17β4*), *glucocorticoid receptor *(*NR3C1*), *oxysterol binding protein *(*OSBP*), and *retinoic acid receptor α *(*RARA*). The observation of functionally related groups of genes identified in the SAGE dataset via GO over representation analysis allows the identification of distinct biological pathways directly or indirectly associated to estrogen response related processes and provides the basis for future mechanistic studies.

### Identification of high-affinity Estrogen Response Elements

**Figure 3:**
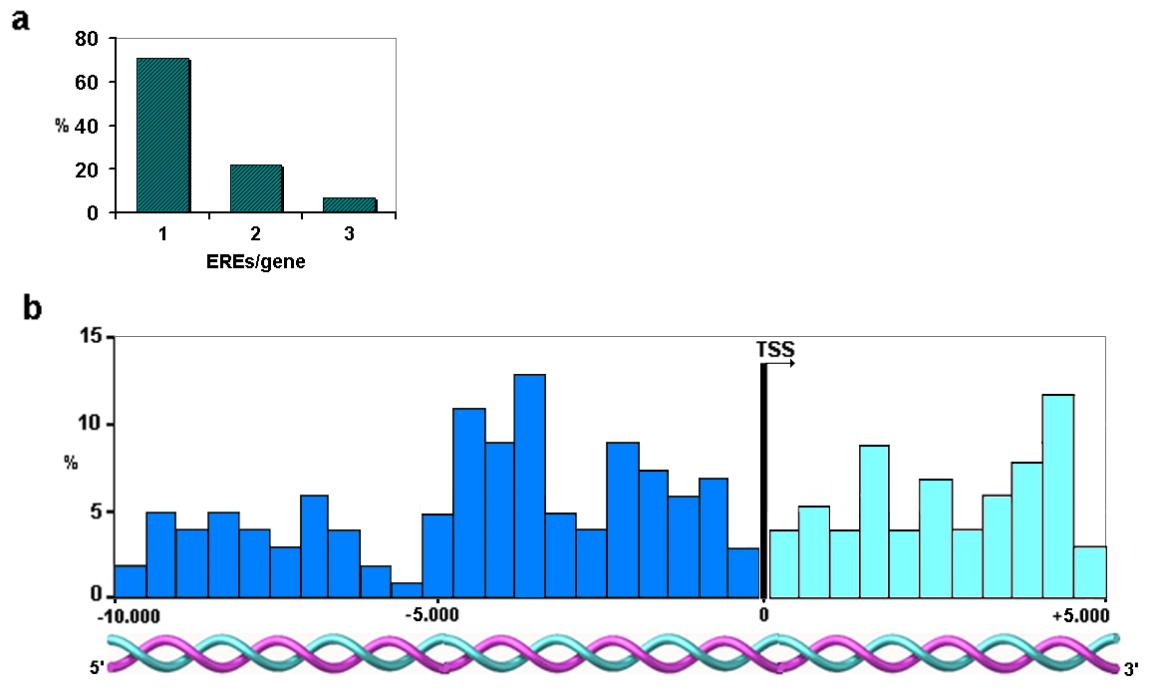
**High-affinity EREs in ERα (+) up-modulated genes (n = 163). a) **Percentage of genes according to number of EREs. **b) **Distribution of EREs in 5' (blue bars) and 3' (aquamarine bars) regions relative to the TSS (-10 to + 5 kb). Each bar represents an interval width of 500 bp.

We used a recently reported genome-wide high-affinity ERE database [32] to identify putative EREs in the promoter regions of the SAGE-identified 473 up-modulated genes in ERα (+) breast tumors. We identified 220 EREs distributed on 163 out of the 473 genes (35%) (see additional data file 3). Seventy-two percent of these genes contain one high affinity ERE (117 out of 163) and 28% of them contain two or more EREs in proximity to the transcriptional start sites (TSS) (46 out of 163) (Figure 3a). These EREs can be located in both coding and non-coding sequences such as was described by Bourdeau *et al*. [32].

The observed frequency of these elements in our study was 220 EREs in 3260 kb (considering a DNA window of 20 kb for each one of the 163 up-modulated genes with EREs). Compared with the expected frequency from random distribution of high-affinity EREs found in the genome (732 EREs in 3,069334 kb 0.8 ERE in 3260 kb) (see material and methods) [32], the number of individual EREs was 270 fold higher than expected by chance (p < 0.00001).

Fifty percent (110 out of 220) of the detected EREs mapped within a 10 kb region 5' of the TSS, while the rest mapped to 3' regions (Figure 3b). Approximately 68% of EREs mapped within the region between -5 to +5 kb from the TSS; in agreement with those observations of Bourdeau *et al*. [32]. However, it remains to be determined whether distantly located EREs (e.g. -10 kb from the TSS) are functional E_2_-ER binding sites related to transcriptional activation.

Of the validated transcripts previously discussed (Figure 1), we detected high-affinity EREs on the upstream or downstream regions related to the TSS of *SYTL4 *(-8384 bp from the TSS: tggacatcatgacct), *TSPAN1 *(+974 bp and +9384 bp from the TSS: tggtctgaatgaccc and aggtcatttccacct respectively), *CELSR2 *(+173 bp and +3607 bp from the TSS: tgctcagggtgaccc and aggtcaccatgaccg respectively), and *NR4A1 *(-3478 bp and +4217 bp from the TSS: tgttcactctgacct).

It is interesting to note that we were unable to identify high-affinity EREs on the majority of deregulated genes (65%) associated with a positive ER α status. The possibility exists that many of these genes are transcriptionally regulated by non-ERE mediated mechanisms such as those involving ER binding to the AP1 or SP1 transcription factors [33]. The AP1 transcription factor is a heterodimer formed by Jun and Fos family member proteins that binds to the phorbol diester (TPA) response element as well as to the AP1 consensus DNA sequence. In this pathway, ER plays a co-activator role for AP1 [6]. The ER/AP-1 complex can confer estrogen responsiveness to additional subset of genes found in our dataset such as: *ovalbumin *(Fold change: 3; p = 0.033) and *c-fos *(Fold change: 2.1; p = 0.033); two transcripts detected as over-expressed in ERα (+) breast tumors by SAGE (additional data file 1). Similarly the ER/SP1 complex confers estrogen responsiveness to genes such as: *retinoic acid receptor α *(*RARA*) (Fold change: 6.7; p = 0.038), *vascular endothelial growth factor *(*VEGFC*) (Fold change: 2.6; p = 0.037), *insulin-like growth factor binding protein-4 *(*IGFBP4*) (Fold change: 2; p = 0.01) and *heat shock protein 27 *(*HSPB1*) (Fold change: 2; p = 0.045); four transcripts detected as over-expressed in ERα (+) tumors in our study (additional data file 1).

An additional pathway of transcription regulation by estrogen involves the ER-related receptors (ERR), *nuclear orphan receptors *with significant homology to ERs, which do not bind estrogen and have unknown physiological ligands. ERRs are known to bind to the steroidogenic factor 1 response element (SFRE) and also bind to classic EREs, by means of which they exert constitutive transcriptional activity [34]. We detected over-expression of the nuclear orphan receptor *NR4A1 *by SAGE and subsequently validated this observation by real time RT-PCR (Figure 1g). Interestingly, and as previously mentioned, the genomic region 5' and 3' to the TSS of *NR4A1 *contain high-affinity EREs. Interaction between ERs and ERRs has been observed in the transcriptional regulation of certain genes such as the human breast cancer related gene TFF1/pS2, the promoter of which is not only activated by ERs but also by ERRs [35].

As described, ERα can mediate estrogenic response through multiple genomic and non-genomic mechanisms, many of which affect proteins and pathways not necessarily directly or exclusively associated with ERα. Thus it is worth stressing that it will the totality of deregulated proteins the ones that ultimately define the phenotype of ERα (+) breast carcinomas regardless of whether a "direct association" with ER transcriptional regulation exists or not.

### *In vivo *versus *in vitro *estrogen induced global gene expression findings

The SAGE profiles for E_2_-responsive genes in MCF-7 cell line, previously reported by us [36], was compared with the ER status genes expression profile found in primary breast carcinomas. Briefly, we detected 199 transcripts differentially expressed (p < 0.01) in MCF-7 treated cells, 124 were up-regulated and 75 were down-regulated transcripts. Basically and as reported Charpentier *et al*, we observed a general up-regulation cell cycle progression-related genes including: *CCT2*, *CCND1*, *PES1*, *RAN/TC4*, *CALM1*, *CALM2*; and tumor-associated genes such as: *RFP*, *D52L1*, *TFF1/PS2*, *CAV1*, and *NDKA *among others [36]. These together could contribute to the stimulation of proliferation and the suppression of apoptosis by E_2_-ER transcriptional regulation.

By comparing the *in vitro *(199 differentially expressed transcripts) and *in vivo *(520 differentially expressed transcripts) gene expression profiles, to our surprise we detect that only few transcripts: *TFF1, CCND1, H19, SREBF1 *and *WWP1 *behaved similarly (i.e. up-regulation) in both studies. This is similar to observations made previously by Meltzer and co-workers whom showed that the majority of genes regulated in cell culture do not predict ER status in breast carcinomas [11,37]. This result suggests that the estrogen-responsive pathways affected in vitro represent only a minor portion of the global gene expression profiles characteristic of ERα (+) breast tumors. This maybe in great part the result of the heterogenous nature of bulk tumor tissue but in addition, the *in vitro *response of a single cell line to E_2_, in this particular case the widely used MCF-7 cells, may not faithfully reproduce the physiological effects of ER signaling *in vivo*.

### Cross-platform gene expression profiling comparison

In order to identify and validate the most reliable set of genes able to discriminate breast carcinomas based on their ERα status, we performed a cross-platform comparison between the described SAGE dataset with two previously reported breast cancer studies based on DNA microarray methods [12,13]. van't Veer *et al. *[12] reported the gene expression profile of 97 primary breast tumors based on oligonucleotide microarrays containing 24,479 elements (Agilent Technologies, Palo Alto, CA, USA). In another study, Sotiriou *et al. *[13] reported the gene expression profile of 99 primary breast tumors using a cDNA microarray containing 7650 elements. Only files containing differentially expressed genes associated to ERα status tumors from both microarrays studies were obtained for cross-platform comparison (see material and methods).

**Figure 4:**
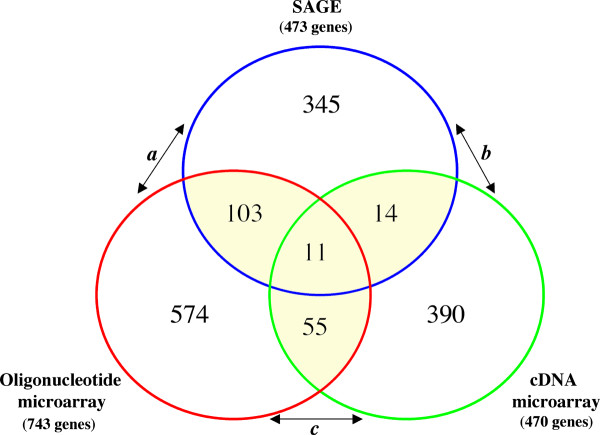
**Cross-platform comparisons of the up-modulated transcripts in ERα (+) breast carcinomas. **One hundred and eighty-three genes were identified by more than one study, eleven of which were commonly identified across the three platforms. **a) **Comparison between SAGE and oligonucleotide microarray platforms [12] showing a highly significant number of overlapping genes (p < 0.001) (see table 2). **b) **Comparison between SAGE and cDNA microarray platforms [13] (p > 0.05). **c) **Statistically significant number of overlapping genes identified by both DNA microarrays platforms (p < 0.01).

Among the three platforms, a total of 1686 transcripts were identified as over-expressed in ERα (+) breast tumors. One hundred and eighty-three genes were identified by more than one method (Figure 4; additional data file 4). Eleven of these 183 genes were identified by all three methods displaying over-expression in ERα (+) breast carcinomas: *estrogen receptor 1 *(*ESR1*), *GATA-binding protein 3 *(*GATA3*), *mucin 1 *(*MUC1*), *v-myb-myeloblastosis viral oncogene homolog *(*C-MYB*) , *X-box-binding protein 1 *(*XBP1*), *hydroxysteroid 17-β dehydrogenase 4 *(*HSD17B4*), *BTG family member 2 *(*BTG2*), *transforming growth factor β-3 *(*TGFB3*), *member RAS oncogene family *(*RAB31*), *START domain containing 10 *(*STARD10*), and *KIAA0089 *(Table 2).

**Table 2:** Transcripts identified as over-expressed in ERα (+) breast cancers commonly detected by cross-platforms comparison (SAGE and oligonucleotide microarrays).

**Gene name**	**Locus Link ID**	**Fold change**	**Frequency**	**Gene name**	**Locus Link**	**Fold change**	**Frequency^#^**
TFF1*	7031	51.4	15/18 (83%)	***SULF2***	55959	2.9	11/18 (61%)
SYTL4*	94121	28.0	15/18 (83%)	***THBS4***	7060	2.9	8/18 (44%)
***DUSP4***	1846	14.7	14/18 (78%)	***AZGP1***	563	2.8	9/18 (50%)
***NAT1***	9	11.7	15/18 (83%)	BBC3*	27113	2.8	12/18 (67%)
ECM1*	1893	10.1	13/18 (72%)	NET7*	23555	2.8	10/18 (55%)
***KIAA0882***	23158	10.1	18/18 (100%)	***NET6***	27075	2.8	12/18 (67%)
***JDP1***	56521	10.0	12/18 (67%)	***TRAF5***	7188	2.8	9/18 (50%)
***ESR1***	2099	9.8	18/18 (100%)	***BTG2***	7832	2.7	9/18 (50%)
***HDGFRP3***	50810	9.8	12/18 (67%)	RNF123*	63891	2.7	11/18 (61%)
***TCEAL1***	9338	9.8	13/18 (72%)	CHAD*	1101	2.6	12/18 (67%)
TSPAN1*	10103	9.5	15/18 (83%)	***CSNK1A1***	1452	2.6	14/18 (78%)
C20orf103*	24141	9.3	10/18 (55%)	***EVL***	51466	2.6	12/18 (67%)
***MYLIP***	29116	9.3	11/18 (61%)	***HIST1H2BD***	3017	2.6	10/18 (55%)
***ABCA3***	21	8.9	10/18 (55%)	***SUSD3***	203328	2.6	9/18 (50%)
***SEC14L2***	23541	8.7	9/18 (50%)	PLAT*	5327	2.6	8/18 (44%)
ANXA9*	8416	8.4	10/18 (55%)	RARRES3*	5920	2.6	11/18 (61%)
***KCTD3***	51133	8.4	17/18 (94%)	SH3BGRL*	6451	2.6	8/18 (44%)
***SCUBE2***	57758	7.5	14/18 (78%)	TPBG*	7162	2.6	9/18 (50%)
***ITGBL1***	9358	7.1	8/18 (44%)	***UGCG***	7357	2.6	11/18 (61%)
***C14orf168***	83544	6.7	6/18 (33%)	CELSR2*	1952	2.5	8/18 (44%)
***FBP1***	2203	6.7	14/18 (78%)	***CRIM1***	51232	2.5	11/18 (61%)
***MYB***	4602	6.7	14/18 (78%)	FLJ90798*	219654	2.5	9/18 (50%)
RARA*	5914	6.7	12/18 (67%)	***KIF12***	113220	2.5	7/18 (39%)
***CaMKIINα***	55450	6.3	18/18 (100%)	***LRIG1***	26018	2.5	9/18 (50%)
AR*	367	6.2	10/18 (55%)	LRP2*	4036	2.5	10/18 (55%)
***ZNF552***	79818	6.2	16/18 (89%)	PHF15*	23338	2.5	12/18 (67%)
MIPEP*	4285	6.0	14/18 (78%)	***HSMNP1***	55861	2.4	8/18 (44%)
***BAI2***	576	5.3	15/18 (83%)	***LOC123169***	123169	2.4	12/18 (67%)
***DP1L1***	92840	5.3	15/18 (83%)	PINK1*	65018	2.4	11/18 (61%)
***VAV3***	10451	5.3	12/18 (67%)	***PRKAR2B***	5577	2.4	7/18 (39%)
***KIAA0089***	23171	5.2	17/18 (94%)	TJP3*	27134	2.4	11/18 (61%)
***GATA3***	2625	5.1	15/18 (83%)	***CCND1***	595	2.3	9/18 (50%)
***QDPR***	5860	5.1	11/18 (61%)	***CYBRD1***	79901	2.3	10/18 (55%)
***C1orf21***	81563	4.9	11/18 (61%)	***KRT18***	3875	2.3	10/18 (55%)
***KIAA1143***	57456	4.9	7/18 (39%)	***PURA***	5813	2.3	9/18 (50%)
***OIP106***	22906	4.9	16/18 (89%)	SREBF1*	6720	2.3	10/18 (55%)
***AGR2***	10551	4.6	10/18 (55%)	***CYB5R1***	51706	2.2	6/18 (33%)
***MGC4251***	84336	4.6	13/18 (72%)	DLG3*	1741	2.2	9/18 (50%)
***FER1L3***	26509	4.4	10/18 (55%)	***EEF1A2***	1917	2.2	11/18 (61%)
***C4A***	720	4.1	11/18 (61%)	***GSTZ1***	2954	2.2	9/18 (50%)
***CRIP2***	1397	4.0	15/18 (83%)	***LOC159090***	159090	2.2	6/18 (33%)
***NTN4***	59277	4.0	10/18 (55%)	MGC11242*	79170	2.2	10/18 (55%)
***GJA1***	2697	3.8	11/18 (61%)	MGC18216*	145815	2.2	8/18 (44%)
CGI-111*	51015	3.7	14/18 (78%)	***NEIL1***	79661	2.2	6/18 (33%)
CROT*	54677	3.6	15/18 (83%)	XBP1*	7494	2.2	8/18 (44%)
***DACH***	1602	3.6	13/18 (72%)	***IRX5***	10265	2.1	8/18 (44%)
***DKFZP564D172***	83989	3.6	10/18 (55%)	***RAB31***	11031	2.1	9/18 (50%)
***FGD3***	89846	3.6	10/18 (55%)	***SSBP2***	23635	2.1	7/18 (39%)
RNASE4*	6038	3.6	12/18 (67%)	***TGFB3***	7043	2.1	8/18 (44%)
GLUL*	2752	3.3	11/18 (61%)	***BMPR1B***	658	2.0	7/18 (39%)
***FOXA1***	3169	3.2	10/18 (55%)	***FLJ21174***	79921	2.0	6/18 (33%)
***MGC7036***	196383	3.2	14/18 (78%)	***FLJ22386***	79641	2.0	7/18 (39%)
MUC1*	4582	3.2	12/18 (67%)	HSPB1*	3315	2.0	6/18 (33%)
***NAV1***	89796	3.1	13/18 (72%)	IGFBP4*	3487	2.0	8/18 (44%)
RPLP1*	6176	3.1	12/18 (67%)	MGC15737*	85012	2.0	8/18 (44%)
***ALCAM***	214	2.9	9/18 (50%)	***SPARCL1***	8404	2.0	9/18 (50%)
HSD17B4*	3295	2.9	13/18 (72%)	STARD10*	10809	2.0	7/18 (39%)

* Genes with known or putative high-affinity EREs mapping in the vicinity of the TSS.

^# ^Transcripts tags changing > 2-fold when compared with the average expression of ERα (-) tumors. Underlined genes correspond to the transcripts cross-validated among all three compared platforms.

One hundred and fourteen genes were identified as over-expressed by oligonucleotide microarrays [12] and SAGE in ERα (+) tumors, representing a non-random significant number of overlapping genes based on normal approximation to the binomial distribution (p < 0.001) (Figure 4 and Table 2). Sixty-six genes were identified as over-expressed in ERα (+) tumors by both DNA microarrays platforms (p < 0.01). The set of 25 genes overlapping between cDNA microarrays [13] and SAGE were not statistical significant (p > 0.05).

Interestingly, we found a higher number of overlapping genes between the oligonucleotide microarray and SAGE platforms (114 genes), while only 66 genes were observed overlapping when comparing both microarray platforms. It is worth noting that 96% of the 470 genes (Figure 4) identified as overexpressed by the cDNA microarray method [13] were included within the total set of elements in the oligonucleotide microarray platform [12]. In other words, it appears that a better correlation was observed between SAGE and oligonucleotide arrays, than between both DNA microarray methods.

## Conclusion

In summary, our comprehensive comparison of overlapping genes across different gene expression platforms provides validation for a significant number of transcripts identified as highly expressed in ERα (+) breast tumors. More importantly this analysis identifies the most promising biomarkers for further evaluation as ERα associated genes in breast cancer. Furthermore, the identified proteins may be of value as breast cancer prognostic indicators analyzed either as a group or individually. It is also likely that groups of co-regulated genes in ERα (+) breast cancers may be associated to the hormonal control of mammary epithelial cells growth and differentiation. Finally, a better understanding of the signaling networks controlled or associated with the estrogen response may lead to the identification of novel breast cancer therapeutic targets.

## Methods

### SAGE libraries

To perform the comparative breast cancer SAGE analysis based on ERα status, we analyzed 26 Stage I – Stage II invasive breast carcinomas (8 ERα-negative tumors and 18 ERα-positive tumors). To this end, we generated and sequenced 24 breast cancer SAGE libraries at an approximate resolution of 100,000 tags per library, combined with 2 additional breast cancer libraries (ERα-negative tumors) downloaded from the Cancer Genome Anatomy Project – SAGE Genie database (SAGE_Breast_Carcinoma_B_95-259 and B_IDC_4) . For the generation of our SAGE libraries, snap frozen samples were obtained from the M.D. Anderson breast cancer tumor bank, and SAGE analysis was performed as previously described [36,38].

### Data processing and statistical analysis of SAGE libraries

SAGE tag extraction from sequencing files was performed by using the SAGE2000 software version 4.0 (a kind gift of Dr. K. Kinzler, John Hopkins University). SAGE data management, tag to gene matching as well as additional gene annotations and links to publicly available resources such as GO, UniGene, LocusLink, were performed using a suite of web-based SAGE library tools developed by us . In our analyses we only considered tags with single tag-to-gene reliable matches. To compare these SAGE libraries, we utilized a modified t-test recently developed by us [18]. This test is based on a beta binomial sampling model that takes into account both, the intra-library and the inter-library variability, thus identifying 'common patterns' of SAGE transcript tag changes systematically occurring across samples [18].

All raw SAGE data reported as Supplementary tables in this manuscript is publicly available at .

### Real Time RT-PCR analysis

Template cDNAs were synthesized on mRNAs isolated from an independent set of 36 Stage I – Stage II human breast carcinomas (13 ERα-negative tumors and 23 ERα-positive tumors) obtained from our tumor bank. Primers and probes were obtained from the TaqMan Assays-on-Demand™ Gene Expression Products (Applied Biosystems, Foster City, CA, USA). All the PCR reactions were performed using the TaqMan PCR Core Reagents kit and the ABI Prism^® ^7700 Sequence Detection System (Applied Biosystems, Foster City, CA, USA). Experiments were performed in duplicate for each data point and 18s rRNA was used as control. Results were expressed as mean ± 2 Standard Error based on Log2 transformation of normalized real time RT-PCR values of the assayed genes. We used t-test to compare the gene expression levels of validated genes between ERα (+) and ERα (-) breast tumors (p < 0.05).

### Immunohistochemical determination of ER status

IHC staining and ER status determination was performed by the Pathology Department, MDACC following routine immunohistochemical procedures. Briefly, five micrometer sections of invasive breast carcinomas paraffin embedded tissues were used. Endogenous peroxidase activity was blocked with 3% H_2_O_2 _in methanol for 10 min. After pretreatment with Tris-EDTA buffer, in order to block non-specific antibody binding, the slides were incubated with 10% goat serum in PBS for 30 min. Primary monoclonal ERα antibody (ER-6F11, Novocastra, Newcastle, UK) was used at 1:50 dilution and detected following standard immunohistochemical techniques. DAB was used as chromogen and Mayers hematoxylin is used as counterstain. Scoring was performed by breast pathologist (AS). Cuttoff for positivity was determined at 5% of tumor cells staining positively for ER (i.e. < 5% of cells the tumor was considered negative for ERα).

### Bioinformatics analysis

For automated functional annotation and classification of genes of interest based on GO terms, we used the *EASE *[31] available at the *Database for Annotation, Visualization and Integrated Discovery *(*DAVID*) at [39]. The *EASE *software calculates over-representation of specific GO terms with respect to the total number of genes assayed and annotated. Statistical measures of specific enrichment of GO terms are determined by means of an *EASE *score (p < 0.05). The *EASE *score is a conservative adjustment of the Fisher exact probability that weights significance in favor of biological themes supported by more genes and is calculated using the Gaussian hypergeometric probability distribution that describes sampling without replacement from a finite population [31]. This allows one to identify biological themes within a specific list of *EASE *analyzed genes.

### High-affinity Estrogen Response Elements (ERE) analysis

To identify the occurrence of EREs within the promoter regions of up-modulated genes in ERα (+) breast tumors, we used a human genome-wide high-affinity ERE database [32]. This public available database contains 71,119 EREs identified across the human genome (related to 17,353 transcriptional start sites), representing the consensus ERE (5'-Pu-GGTCA-NNN-TGACC-Py-3'), and equivalent sequences with only one or two nucleotide variations from such consensus. Based on these restrictions the expected random frequency was calculated as the total number of base pairs in the human genome divided by the frequency of occurrence of a sequence with specified base pairs at 10 positions and two base pair choices at two positions (3,069334246/4^11 ^= 732 high-affinity EREs) [32].

### Comparison of gene expression patterns identified by different methodologies

ERα status associated genes identified in previous breast cancer studies [12,13] using DNA microarray methods were compared with our SAGE findings.

All over-expressed genes in ERα (+) breast tumors obtained from these studies were downloaded from the corresponding web sites ( and ) [12,13].

These datasets were annotated by LocusLink ID using the *EASE *software [26], and then compiled into one Excel spreadsheet pivotTable for comparison of overlapping genes between platforms, i.e. SAGE, Oligonucleotide and cDNA arrays. Anonymous ESTs from the microarrays platforms were excluded due to the inability to cross validate the identities between different gene expression profiles. Any combination of two lists was compared for matching gene-identity. The number and identity of genes commonly affected in two platforms (*e.g. *SAGE study vs. DNA microarray) was determined. We used the normal approximation to the binomial distribution as previously described [40] to calculate whether the number of matching genes derived from each cross-platform comparison was of statistical significance (p < 0.05).

## Authors' contributions

**M.C.A. **conceived the study idea and carried out the real time RT-PCR validations, the biostatistical/ bioinformatics analysis and writing the manuscript. **Y.H.**, **H.S. **and **J.A.D. **carried out the breast cancer SAGE libraries and provided practical feedback on aspects of the manuscript. **K.B. **and **S.G. **developed the biostatistical and web-page base methodology. **A.S. **provides the tissue samples and clinical information. **C.M.A. **is the principal investigator and was involved in the conceptualization, design and writing of the manuscript. All authors read and approved the final manuscript.

## Competing interests

The author(s) declare that they have no competing interests.

## Supplementary Material

Additional File 1 Differentially expressed genes between ERα (+) vs. ER α (-) breast carcinomas (Fold change ≥2; p < 0.05). Click here for file

Additional File 2 Gene Ontology overrepresentation analysis. Click here for file

Additional File 3 High-affinity EREs identified in ERα (+) up-modulated genes. Click here for file

Additional File 4 Cross-platform comparison of the up-modulated transcripts in ERα (+) breast carcinomas. Click here for file
